# Exploration of college teachers’ psychological adaptation to online teaching during the COVID-19 pandemic using potential profile analysis

**DOI:** 10.1371/journal.pone.0278896

**Published:** 2022-12-12

**Authors:** Weixing Zou, Xiangmei Ding, Hongli Wang

**Affiliations:** 1 School of Educational Science, Minzu Normal University of Xingyi, Xingyi, China; 2 School of Psychology, Guizhou Normal University, Guiyang, China; Universidade do Vale do Rio dos Sinos, BRAZIL

## Abstract

The present study used a person-centered approach to examine college teachers’ psychological adaptation to online teaching and its relationship with demographic variables. A total of 2104 college teachers were surveyed using the Psychological Adaption to Online Teaching Scale between March 25 and March 31, 2020. Data were analyzed using latent profile analysis, chi-square test, and multinomial logistic regression analysis. Based on their psychological adaptation during online teaching immediately after the coronavirus disease 2019 (COVID-19) outbreak, college teachers were divided into three latent profiles: common, maladaptive, and positive. Among these, the common type accounted for the largest proportion (56.1%), while the maladaptive type accounted for the smallest (10.9%). There were significant differences in the distribution of psychological adaptation latent types in college teachers with different educational backgrounds and professional titles. A better educational background and higher professional title is closely related with college teachers’ psychological adaptation to online teaching.

## Introduction

The global outbreak of the coronavirus disease 2019 (COVID-19) pandemic has had a major impact on all aspects of people’s daily lives. By the end of June 2020, more than 1 billion learners had been affected by the closure of schools, colleges, and universities due to COVID-19 [1]. The outbreak led to higher education institutions transitioning to online teaching. In response to the pandemic, traditional classroom teaching was promptly transferred to online teaching [2]. Without prior exposure to online teaching and technology-related training, teachers had to quickly adjust their courses and methods, thus posing a huge challenge to teachers at all levels, from elementary school to university education [3, 4].

Although large-scale online teaching in China has achieved and even exceeded expectations, there remain some problems at the teacher level with the novel situation [5]. Despite this, most studies on the pandemic have focused on the psychological state of students and the public [6–8]. Few studies in China have focused on the psychological state (i.e. anxiety, stress) of teachers during the pandemic [9, 10]. Where studies did focus on teachers, they were based on a variable-centered perspective. To the best of our knowledge, there is no research that specifically explores teachers’ psychological adaptation and development owing to the novelty and challenge of online teaching from home. Therefore, this study aims to investigate the psychological stress experiences and adaptation characteristics of college teachers with respect to online teaching.

Psychological adaptation refers to an individual’s positive response to changes in their external environment, and adapting their mentality and behavior patterns to environmental changes and developmental needs to achieve a balance between themselves and the environment [11]. Previous studies have used indicators, such as loneliness, depression, anxiety, and self-esteem, as reflections of psychological adaptation [12–14]. In addition to explaining differences in individual performance, Lazarus’ stress and coping model theory provides a theoretical framework for individuals to maintain their mental health under pressure [15]. According to this theory, individual resources, situational attributions, cognitive evaluation, and coping strategies are some of the important factors affecting an individual’s adaptation. In addition, the holistic person-context interaction theory points out that individuals achieve positive psychological adjustments in two ways, namely: internal interaction (individuals’ physiological, psychological, and behavioral interaction) and external interaction (between the individual and their environment) [16].

As COVID-19 prevention and control policies continue, teachers who have switched to online teaching with little training may experience, in some cases, increased stress levels [15]. A large amount of teachers’ work (i.e., lecture preparation, the content to be taught) has been complicated by the rapid transformation to online delivery. Balancing personal and professional life is a challenge for many teachers. The provision of ubiquitous online teaching and student education at home has simultaneously affected teachers’ school and family responsibilities. The lack of physical, temporal, and psychological boundaries between work and family can cause serious psychological conflict and discomfort. In many cases, teachers not only need to share the same space with important family members to carry out their work, but their children also need their attention. In addition, there are concerns regarding personal and family members’ health as social distancing and daily life services are limited, and the uncertainty of when life will return to normal, may affect teachers’ psychological balance [17]. Therefore, the severe situation brought about by the pandemic and the circumstances of home online teaching will likely have an important impact on teachers’ psychological adaptation to online teaching.

Teachers’ psychological adaptation to online teaching as explored in this study refers to the closure of face-to-face courses and the sudden switch to online courses. Teachers had to find innovative teaching and evaluation methods for the continuation of students’ learning. College teachers had to align their cognition and behaviors with this new teaching method and environment to achieve a balance between both of them. Online teaching is a challenging and relatively new field of teaching. College teachers’ psychological experiences during the pandemic have been complex and diverse. College teachers need to think, learn, and implement online teaching in a creative manner to better adapt to this challenging task [5]. Therefore, the development of college teachers’ psychological adaptations to online teaching is worthy of discussion.

In summary, since college teachers had to promptly switch to online teaching during the COVID-19 pandemic, it is important to explore whether or not they could quickly adapt to this challenge. Thus, this study explores the potential types of psychological adaptations that might exist for college teachers during the pandemic. Further, it aims to address the following questions: What is the percentage of teachers in different categories? What are the work experiences and adaptation results produced in different categories? These issues are pertinent but difficult to explore further with a variable-centric perspective, which has been used in past studies. Therefore, we use a different approach to explore this topic.

Latent profile analysis (LPA) is a statistical method that explains the relationship between exogenous continuous-type indicators through latent category variables, so that associations between exogenous indicators are estimated through latent category variables, thus maintaining local independence among exogenous indicators. The underlying assumption is that a small number of mutually exclusive latent category variables, each of which has a specific tendency to select responses to the exogenous variables, can explain the probability distributions of the responses of the exogenous variables [18].

In the field of clinical psychology, latent category/profile analysis classifies clinical samples into different subgroups based on symptoms or psychopathological features to explore specific comorbidity patterns [19].

Researchers can use this method to understand how different variables are combined and attribute the results to specific groups. In other words, different types of subgroups can be identified based on the attributes and ranges of the explicit variables, which helps to check for intragroup heterogeneity that cannot be observed in variable-centric studies [20]. A past study using LPA identified three potential teacher stress types, namely distressed (helplessness), moderate stress, and positive stress (self-efficacy). High helplessness and low self-efficacy scores characterize the distressed type, while low helplessness and high self-efficacy characterize the positive stress type [21]. LPA is a good method for identifying different teachers’ stress types based on teachers’ self-reported perceived stress.

To the best of our knowledge, no analysis, based on an individual-centered perspective, has been done on individuals’ psychological characteristics during the COVID-19 pandemic. This is particularly true for teachers’ characteristics and psychological adaptation to complex tasks in the early stage of online teaching. Therefore, this study focused on the psychological adaptation of college teachers engaged in online teaching during the pandemic, and independently used the specific manifestations of online teaching psychological adaptation as indicators. Further, LPA was used to explore the specific heterogeneous characteristics and conditions of college teachers’ psychological adaptation to online teaching during the COVID-19 pandemic.

## Materials and methods

### Sampling and participants

This study used a convenience sampling method. A total of 2197 college teachers from multiple provinces were recruited to participate in online questionnaire surveys on the "Questionnaire Star" online survey platform(https://www.wjx.cn/) from March 25 to March 31, 2020. Previous studies have found that overly quick response times can be used as an important indicator for judging invalid questionnaires [22]. After the questionnaires were checked for their validity, 2104 valid questionnaires were obtained, resulting in an effective response rate of 95.8%. Among these, there were 878 males and 1226 females; 278 professors, 708 associate professors, 769 lecturers, and 349 teaching assistants; 1058 were in the social sciences, 729 were in science and engineering, and 317 were in arts and sports. The college teachers’ ages ranged from 20 to 69 years, and their teaching experience was between 0.5 and 40 years. Informed consent was obtained from all participants involved in the study. Ethical approval for the study was obtained from the Research Ethics Committee of the School of Education Science, Xingyi Normal University for Nationalities (No.ESXYNUN20200315).

### Research tools

#### Psychological adaptation scale of online teaching for college teachers

This scale comprises of positive and negative adaptations. Six items from the positive adaptation dimension (for example, “I believe I am competent for online teaching,” and “I can do well in every online class as long as I work hard”), and eight items from the maladjustment dimension (for example, “If I have an online course to teach the next day, I will worry about the poor effect and quality of the online course and not sleep well”) formed a total of 14 items. Five-point Likert-type scales (1 = never, 5 = always) were used for all measurements. The reliability test of the scale showed that the α reliability coefficient of the positive adaptation dimension was 0.737, and the α reliability coefficient of the maladaptive dimension was 0.835, indicating that the consistency of the two aspects of the scale was high [23].

#### Demographic variables of college teachers

The demographic variables included sex (male, female), age, teaching experience, highest completed education level (undergraduate, masters, doctorate), professional title (professor, associate professor, lecturer, and assistant), disciplines (social sciences, science and engineering, arts and sports), and type of school (ministerial universities, provincial key universities, provincial general universities, municipal universities, and higher vocational colleges).

### Statistical analysis

Mplus 7.4 software was used to analyze and to judge the potential profile of all items of the psychological adaptation scale of online teaching for college teachers. SPSS 26.0 software was used to conduct a chi-square test and multinomial logistic regression analysis to explore the relationship between college teachers’ differing demographic information and their psychological adaptation to online teaching.

When analyzing the psychological adaptation profile of college teachers’ online teaching, the number of profiles in the model gradually increased from a two-profile model to the most suitable model. Models’ goodness-of-fit was compared using the following methods [20, 24]: (1) Akaike information criteria (AIC), Bayesian information criteria (BIC), and sample-size adjusted BIC (SSA-BIC); the smaller the index values of these information, the better the fit; (2) Entropy, used to evaluate the accuracy of classification; the larger the entropy, the more accurate the classification; (3) Lo-Mendell-Rubin (LMR) likelihood ratio test and bootstrap likelihood ratio test (BLRT) was used to compare two nested models, a significant p-value (<0.05) of which indicates that k classification models have a better fit than k-1 classification models [25].

## Results

### Potential profile analysis results

Five models with different numbers of latent profiles were estimated and compared, starting with a single-class model and increasing the number of profiles one at a time. The LPA results are presented in Table 1. Based on the number (1 to 5) of potential profiles obtained by the analysis, five models were named, from Model 1 to Model 5. The indicators of each model show that starting from the comparison of the Model 2, as the number of potential profiles increases, the decline in the fitted values of the AIC, BIC, and SSA-BIC to Model 3 starts to decrease. Compared with Model 2, the LMR and BLRT of Model 3 are both significant. In addition, the entropy value of Model 3 is higher than that of Model 2 (0.819 > 0.801), and the highest among all the models. At the same time, compared with Model 3, the LMR and BLRT values of Model 4 are not significant. Although the LMR value of Model 5 is significant, the proportion of each profile is extremely small, and the entropy value of Model 5 is smaller than that of Model 3. Therefore, Models 4 and 5 did not show an effective improvement over Model 3. Considering the simplicity and accuracy of the model, we finally chose three profiles of models as the best fitting model. That is, college teachers are best divided into three potential profiles according to their specific performance of psychological adaptation to online teaching.

**Table 1:** Summary table of fitness indexes to adjusted alternative models of psychological adaptation to online teaching.

Models	AIC	BIC	SSA-BIC	Entropy	LMR(p)	BLRT(p)	Proportion of profiles(%)
Model 1	81824.968	81983.212	81894.253	-	-	-	-
Model 2	77790.615	78033.634	77897.018	0.801	<0.001	<0.001	51.2/48.8
Model 3	76668.127	76995.919	76811.647	0.819	<0.05	<0.001	33.0 /56.1/10.9
Model 4	75733.410	76145.977	75914.049	0.784	0.054	<0.001	12.8/20.7/23.7/42.8
Model 5	75118.767	75616.107	75336.522	0.807	<0.05	<0.001	9.7/13.3/36.0/34.8/6.2

**Fig 1:**
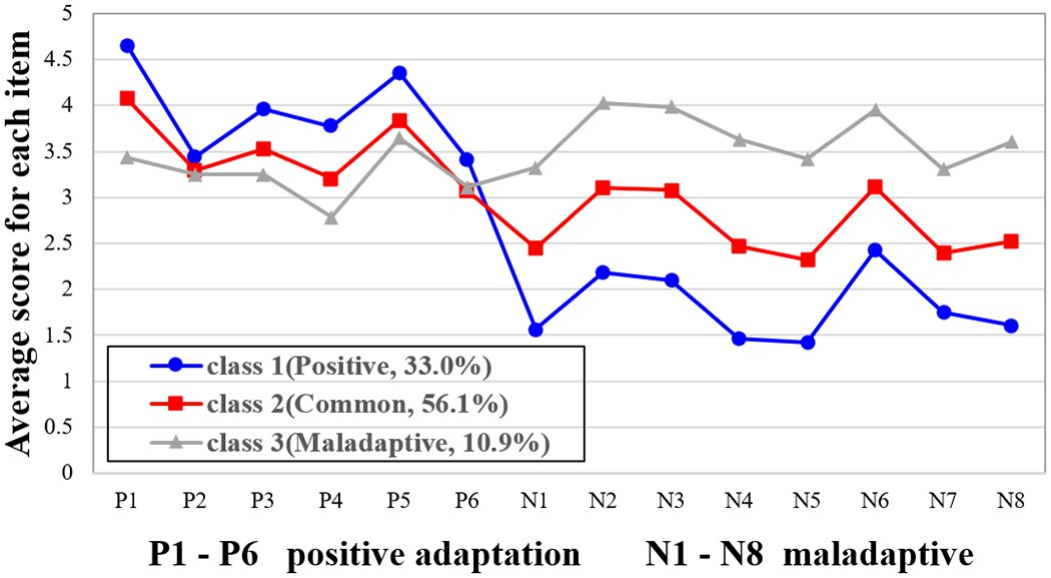
The specific distribution characteristics of various potential profile of college teachers’ psychological adaptation to online teaching.

The specific scores of each item on the two dimensions of college teachers’ psychological adaptation to online teaching in the three potential profile categories are shown in Fig 1. Items P1–P6 in the figure are the positive adaptation dimensions, and N1–N8 are the maladaptive dimensions. Notably, class 1 contains 33.03% of the college teachers. In this class, teachers have the highest positive adaptation and the lowest maladaptation; therefore, this class is named the positive type. Class 3 contained only 10.88% of the college teachers. The college teachers in this class had the highest maladjustment, while their active adaptation was the lowest. Subsequently, the transition trend for class 3 from active adaptation to maladaptation is opposite to that of the other two classes. This class of college teachers is referred to as the maladaptive type. Finally, the proportion of college teachers in class 2 is 56.08%, thereby forming the largest class. In this class, the teachers’ active adaptation and maladaptation are equal in all aspects. Since this was the largest class, it is referred to as the common type.

**Fig 2:**
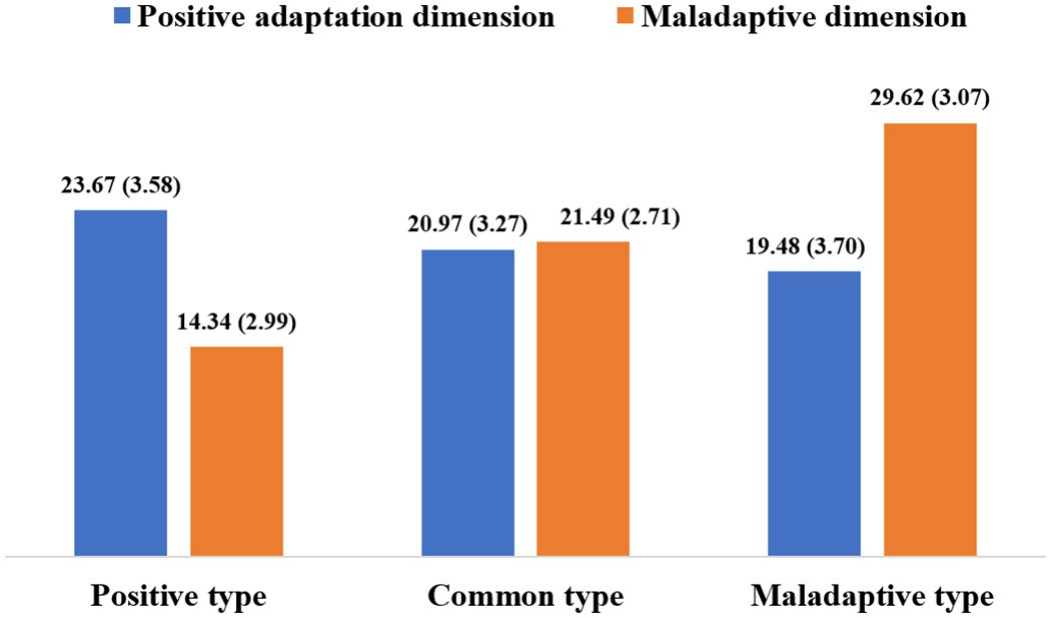
Average score of positive adaptation dimension and maladaptive dimension scores by latent type.

The average bar plots of the scores of the three types of college teachers in the two dimensions of positive adaptation and maladaptive are shown in Fig 2.

### The distribution characteristics of psychological adaptation latent profiles of college teachers in online teaching

The distribution of potential profiles of college teachers’ psychological adaptation in terms of sex, educational background, professional title, and professional discipline is shown in Table 2. The chi-square test in Table 2 shows that teachers with different educational backgrounds and professional titles differ significantly in the latent category distribution of psychological adaptation in online teaching (p < 0.001). College teachers with the highest educational background (doctors) and highest professional title (professors) were the highest proportion of teachers in the positive group. College teaching assistants and teachers with a master’s degree were the highest proportion of teachers in the common group. Associate professors and college teachers with a bachelor’s degree had the largest distribution of teachers in the maladaptive group. These college teachers, relative to college teachers with master’s degrees or doctorate degrees, experienced greater psychological maladjustment in response to online teaching.

**Table 2:** Difference in potential profiles of psychological adaptation by demographics.

Demographic Variables		Positive group	Common group	Maladaptive group	*χ* ^*2*^	*p*
Sex	Male	291(33.14%)	482(54.90%)	105(11.96%)	1.983	0.371
	Female	404(32.95%)	698(56.93%)	124(10.11%)		
Highest education qualification	Doctorate degree	176(38.01%)	239(51.62%)	48(10.37%)	18.588	<0.001
	Master’s degree	350(32.89%)	617(57.99%)	97(9.12%)		
	Bachelor’s degree	169(29.29%)	324(56.15%)	84(14.56%)		
Professional title	Professor	120(43.17%)	129(46.40%)	29(10.43%)	24.488	<0.001
	Associate professor	239(33.76%)	379(53.53%)	90(12.71%)		
	Lecturer	235(30.56%)	456(59.30%)	78(10.14%)		
	Teaching assistant	101(28.94%)	216(61.89%)	32(9.17%)		
Professional discipline	Social sciences	344(32.51%)	591(55.86%)	123(11.63%)	3.586	0.465
	Science and engineering	234(32.10%)	419(57.48%)	76(10.43%)		
	Arts & Physical Education	117(36.91%)	170(53.63%)	30(9.46%)		

### Differences in the demographic characteristics of teachers in different online teaching psychological adaptation subtypes of colleges and universities

Based on the results of the potential profile analysis, this research further explores the demographic differences of college teachers in different online teaching psychological adaptation subtypes. The results of the potential profile analysis were used as dependent variables. Teaching experience, sex (female teacher as a reference point), highest education qualification (Bachelor’s degree as a reference point), professional title (teaching assistant as a reference point), and profession discipline (with Arts and Physical Education as a reference point) were used as independent variables to conduct a multinomial logistic regression analysis. Among them, the maladaptive group was used as a comparative reference category, and the odds ratio (OR) coefficient was obtained. The OR coefficient reflects the odds ratio of teachers’ psychological adaptation to online teaching owing to different teaching experience, sex, highest educational qualification, professional title, discipline and specialty, and college type. The results of the multinomial logistic regression analysis are shown in Table 3.

**Table 3:** Multiple logistic regression analysis of potential profile by college teachers’ demographics.

		Positive type			Common type		
		*B(SE)*	*p*	*OR*	*B(SE)*	*p*	*OR*
Sex	Male	-0.232(0.159)	0.144	0.793	-0.208(0.150)	0.165	0.812
Professional title	Professor	-0.169(0.308)	0.583	1.184	-0.464(0.296)	0.117	0.629
	Associate professor	-0.270(0.245)	0.270	0.763	-0.534(0.229)	0.020	0.586
	Lecturer	-0.147(0.247)	0.550	0.863	-0.224(0.230)	0.330	0.799
Highest education qualification	Doctorate degree	0.744(0.229)	0.001	2.104	0.498(0.217)	0.021	1.646
	Master’s degree	0.682(0.182)	0.000	1.978	0.568(0.170)	0.001	1.764
Professional discipline	Social sciences	-0.577(0.240)	0.016	0.562	-0.313(0.230)	0.175	0.731
	Science and engineering	-0.342(0.248)	0.167	0.710	-0.051(0.238)	0.829	0.950

Note: *B* is the regression coefficient estimates of the results of logistic regression analysis, and SE is the standard errors.

Considering the college teachers from the maladaptive group as a reference, and comparing them with the positive and common groups, the OR results show that compared to maladaptive college teachers, associate professors are more likely to be distributed in the common type than teaching assistants. Teachers in colleges with a doctorate degree or master’s degree are more likely to be distributed among the positive and common types than teachers holding a bachelor’s degree. College teachers in the Arts and Physical Education are more likely to be distributed among the positive type than teachers in the social sciences. Sex and teaching experience have no significant differences in the classification of college teachers’ psychological adaptation to online teaching.

## Discussion

Teaching is usually considered as one of the most stressful occupations, and online teaching during the COVID-19 pandemic poses unprecedented challenges to teachers. Responding to this challenge requires not only knowledge and skills, but also the confidence to successfully carry out online teaching. Psychological characteristics, such as teachers’ self-efficacy and their ability to master modern computer education technology, are the most important resources and factors for teachers’ adaptation to online teaching during the pandemic [26]. All of this indicates that teachers with different individual characteristics and complex environments have greater heterogeneity in their psychological adaptation to online teaching. To accurately understand the characteristics of this heterogeneity, this study adopted an individual-centered research perspective and used LPA to explore the potential types college teachers’ psychological adaptations within one month of the start of online teaching during the pandemic. The results show that there are three different potential types of college teachers’ psychological adaptations in online teaching: the positive, common, and maladaptive type. The positive psychological adaptation group had the best adaptation and the least maladaptation, while the opposite was true for the maladaptive group. The college teachers in the common group had the most common process of adaptation, with both positive adaptation and low maladaptation. From the results of the three potential profiles of college teachers’ psychological adaptation to online teaching in various aspects, we can evidence the complexity of college teachers’ psychological experience of teaching online during the COVID-19 pandemic. There are large individual differences that show that college teachers’ at-home online teaching work will be affected in many ways, which leads to college teachers’ different developmental characteristics, psychological experience, and adaptation during online teaching.

In the face of the novel challenge of online teaching some teachers are able to cope well and realize a positive psychological adaptation, while others may be unable to cope well due to various factors. This study found that the proportion of teachers with maladaptive psychological response was relatively small, perhaps because the pandemic was quickly controlled in China. However, in other countries, some surveys and studies on teachers’ mental health during online learning have shown that teachers have had greater mental health problems during the pandemic compared to pre-pandemic times. For example, social isolation during the pandemic has caused varying degrees of psychological distress to Jordanian university teachers and scientific researchers where college teachers are highly concerned about the COVID-19 virus, social isolation, and the economic impact of the pandemic [27]. During their lockdown period, Israeli teachers were under pressure and had to adapt quickly to provide online teaching. Compared with the normal face-to-face teaching done before the pandemic, after the first week of online teaching, professors’ psychological stress levels increased [28]. A study of Arabic teachers showed that this kind of crises cause teachers to suffer from anxiety, depression, domestic violence, and divorce, thus limiting their ability to teach effectively [29]. Spanish teachers reported an increased workload, physical and mental problems, and exhaustion at the beginning of the pandemic [30].

UNESCO also confirms that the confusion and pressure teachers experience is one of the negative consequences of school closures because these measures are quite sudden, the duration is uncertain, and they are unfamiliar with online education and teaching [31]. Previous studies found that using information and communication technology to work at home can produce feelings of tension, anxiety, exhaustion, and decreased job satisfaction [32]. In addition, teachers’ understanding and emotional attitudes toward the development and changes brought about by the pandemic have become more negative, which may be due to the lack of psychological resources leading to the generation of stress and burnout [33]. Still, during the COVID-19 pandemic, home-based online teaching was the only choice for teachers. As online teaching continues, teachers’ psychological adaptation to online teaching continues to change. Studies have shown that in the first three months of the pandemic, teachers showed greater exhaustion and cynicism, but they also improved their efficiency at classroom management and had a greater sense of accomplishment. This is consistent with the results of this study which show that teachers’ psychological adaptation to online teaching has both positive and negative aspects.

In addition, since teachers’ online teaching work is mainly carried out at home, the psychological adaptation of online teaching is affected by their family environment, with each teacher’s family environment causing them to have differing psychological adaptations to online teaching. For example, some studies suggest that due to the rapid shift to online teaching, the blurred boundary between work and family, and the omnipresent concern for their own health and that of their family, teachers are under high psychological pressure and need to use various skills to handle the pressure [34]. A survey found that teachers who had to take care of their preschoolers and/or young children experienced higher levels of stress [35]. The heavy workload, coupled with the pressure of taking care of the family, may be one of the reasons why teachers with children are under greater pressure, which is also confirmed to a certain extent in the results of the interviews and survey content in this study.

In the face of this challenge, an important aspect of college teachers’ psychological adaptation to online teaching is how to **choose the right coping style** and whether having a reasonable cognition. Teachers in the positive adaptation group may have learned to respond to this stress in an appropriate manner. Studies have shown that after language teachers switched to online teaching during the pandemic, positive mental states (happiness, health, well-being, resilience, and growth during trauma) were positively correlated with teachers’ methodical coping and negatively correlated with avoidance coping. The avoidant coping style is associated with negative outcomes for teachers (stress, anxiety, anger, sadness, and loneliness) [17]. This also indicates that the college teachers in the maladaptive group likely adopt avoidance coping when facing this pressure. The research on Hong Kong teachers during the pandemic shows that challenging events can enhance teachers’ motivation, as they demonstrate a greater commitment to teaching, a strong desire to experience difficulties with their students, and motivate students to strive to equip themselves to cope with uncertain situations [36]. Other studies have found that teachers who teach online experience moderate to high levels of stress, with more than 50% of them spending more than four hours a day on online teaching. The vast majority of them experience technical barriers, but most of them feel that they are able to cope with stress and achieve better adjustment [37].

Difficulties and challenges have a certain impact on individuals, but adopting reasonable coping methods can always result in achieving better adaptation and a normal psychological state. The positive adaptation group in this study reflects this aspect. Although the pandemic resulted in a sudden shift to online teaching which led to a significant decline in teachers’ sense of achievement, teachers who relied on their local and school leaders for strong coordination, targeted training, meaningful collaboration, fair expectations, and recognition for their efforts, have effectively prevented a decline in their sense of achievement [38]. Other research results show that depression and pressure are the main factors affecting online teaching satisfaction, but coping, sources of control, and self-efficacy are important protective factors [39]. At the same time, online teaching remains an important communication method for teacher-student interaction. Teachers in the positive psychological adjustment group may pay more attention to the care of students, social interaction, and student motivation, rather than students’ ability in their subject, which seems to be unique in this novel situation [40]. Further, it also promotes their own positive teaching adaptation. Even though COVID-19 has brought about additional stress and a loss of creativity in most teachers, teachers have great creative adaptability with the support of creative teaching training [41].

Through multinomial logistic regression analysis, this study found that college teachers’ sex and teaching experience had little influence on their psychological adaptation to online teaching, which was somewhat inconsistent with other studies on teachers’ mental health during the pandemic. For example, some studies found that the stress and anxiety levels of female teachers were higher than those of male teachers during the pandemic [34]. Other studies found that female teachers experience significantly more stress during the COVID-19 pandemic, but they often respond to it in a functional way [33]. A study in China assessing the prevalence of anxiety among teachers in the early stages of the pandemic found that women were more anxious than men, and that older teachers had more severe symptoms [9]. The reason for this inconsistency may be that this study is specifically aimed at the psychological experiences and feelings of college teachers in relation to online teaching. Other studies focused more on the overall mental health status of teachers in isolation during the COVID-19 pandemic, thus their results will likely be inconsistent with the results of this study. Meanwhile, both male and female teachers are confronted with this novel challenge and there is little difference in their adaptation to online teaching. This study also shows that there are some specific characteristics in the distribution of psychological adaptation subtypes of online teaching among college teachers with different titles, educational backgrounds, disciplines, and types of colleges. It also shows that the psychological experience and adaptation of college teachers to online teaching during the COVID-19 pandemic is affected by individuals’ different personalities, growth experience, family environment, technology mastery, and so on [15]. This results in the heterogeneity and uniqueness of college teachers’ psychological adaptation to online teaching, which proves the effectiveness of the potential profile classification results obtained by the LPA used in this study.

The value of this study lies in its addressing of the period at the beginning of the COVID-19 pandemic. In adopting an individual-centered research approach, which the authors believe is the first time, this study reveals the complexity of college teachers’ psychological adaptation in the early stage of online teaching. It also reveals the existence of different potential types of college teachers’ psychological adaptation to online teaching using new methods. This study expands the methods, theories, and empirical research in the field of education and teaching, and teachers’ mental health, providing an important basis and reference for the intervention and prevention of physical and mental health problems for college teachers in China. Online education and teaching must involve the cooperation and efforts of the government, schools, teachers, and parents to ensure that teachers and students can improve the efficiency of teaching and learning online.

## Limitations and strengths

One limitation of our study is that it used self-reported data to measure variables, which may potentially influence the reliability of our results through social desirability bias, recall bias, or any other confounding factor. Future research can include different measurement instruments (including observational rating scales) for data collection. Secondly, this study examined the psychological adaptation characteristics of online teaching among college teachers at the individual level. However, individuals’ work is nested in organizations, and future research can analyze at the organizational level within universities and other educational institutions, and use nested cross-level analysis to further explore the heterogeneous characteristics of teachers’ psychological adaptation to online teaching and the related influencing factors at the organizational level.

Thirdly, this study adopts a cross-sectional design. To better understand teachers’ psychological barriers and resilience in education and teaching during the COVID-19 pandemic, future research should investigate the long-term patterns of psychological adaptation to online education and teaching, rather than investigating cross-sectional prevalence. Future study designs should employ forward-looking designs and analyses that integrate multiple risk and resilience factors to improve outcome prediction, whilst taking into account the importance of being flexible as the situation continues to evolve [42].

## Supporting information

S1 Data (SAV)
